# Phenotypic and Genotypic Characterization of Carbapenem‐Resistant *Acinetobacter baumannii* Clinical Isolates: Co‐Existence of *bla*OXA‐23 and *bla*NDM‐1 in a Vietnamese Tertiary Hospital

**DOI:** 10.1155/ijm/7420170

**Published:** 2026-07-29

**Authors:** Tuan Huynh, Quynh Chau, Loan Luong, Kieu Nguyen, Diep Bui, Tinh Pham, Van Pham, My Dung Jusselme

**Affiliations:** ^1^ Department of Microbiology and Parasitology, and Gastro-Hepato Integrated Research Team (GHIRT-002.TCM2025), School of Medicine, University of Medicine and Pharmacy at Ho Chi Minh City, Ho Chi Minh City, Vietnam, hcmut.edu.vn; ^2^ Department of Medical Microbiology, University Medical Center Ho Chi Minh City, Ho Chi Minh City, Vietnam, bvdaihoc.com.vn; ^3^ University of Science Ho Chi Minh City, Ho Chi Minh City, Vietnam; ^4^ Vietnam Research and Development Institute of Clinical Microbiology, Ho Chi Minh City, Vietnam; ^5^ LEESU, Univ Paris Est Creteil, ENPC, Institut Polytechnique de Paris, Creteil, France, u-pec.fr

**Keywords:** *A. baumannii*, *bla*NDM-1, *bla*OXA-23, *bla*OXA-58, CRAB, multiplex real-time PCR

## Abstract

**Background:**

*Acinetobacter baumannii* has emerged as an important opportunistic pathogen due to its increasing resistance to carbapenems, which severely limits therapeutic options. This study is aimed at characterizing the phenotypic resistance profiles and distribution of carbapenemase‐encoding genes among clinical *A. baumannii* isolates collected from a tertiary‐care hospital in southern Vietnam.

**Methods:**

A retrospective study was conducted on 156 *A. baumannii* isolates collected at the University Medical Center Ho Chi Minh City between January and December 2024. Antimicrobial susceptibility testing was performed using the BD Phoenix M50 system. Carbapenemase‐encoding genes, including *bla*OXA‐23, *bla*OXA‐58, *bla*NDM‐1, and *bla*KPC, were detected using multiplex real‐time PCR.

**Results:**

Among the 156 isolates, 70.51% were identified as carbapenem‐resistant *A. baumannii* (CRAB). Most CRAB isolates originated from respiratory specimens (83.64%) and intensive care units (60%). CRAB isolates exhibited extensive multidrug resistance, with resistance rates ranging from 76% to > 96% for most cephalosporins, fluoroquinolones, and aminoglycosides. In contrast, minocycline retained the highest in vitro activity, with a resistance rate of only 10%. The most prevalent carbapenemase gene was *bla*OXA‐23 (73.6%), followed by *bla*NDM‐1 (52.7%) and *bla*OXA‐58 (20.0%). Overall, 95.45% of CRAB isolates were phenotypically identified as carbapenemase‐producing *A. baumannii* (CP‐AB). The co‐occurrence of *bla*OXA‐23 and *bla*NDM‐1 was the most common gene combination, detected in 23.64% of CRAB isolates.

**Conclusion:**

This study demonstrates a high burden of CRAB in a Vietnamese tertiary hospital, largely driven by the co‐existence of *bla*OXA‐23 and *bla*NDM‐1. The identification of carbapenem‐resistant isolates lacking phenotypic carbapenemase activity suggests the involvement of alternative, nonenzymatic resistance mechanisms. Continuous molecular surveillance and strengthened antimicrobial stewardship are urgently needed to limit CRAB dissemination in Vietnam.

## 1. Introduction

The global escalation of antimicrobial resistance (AMR) threatens the effectiveness of antibacterial therapies and significantly increases the risk of treatment failure worldwide [1]. Inappropriate antibiotic use, which accounts for approximately 30% of global consumption and is more prevalent in low‐ and middle‐income countries, is recognized as a key driver of AMR, underscoring the urgent need for standardized surveillance indicators and targeted antimicrobial stewardship interventions [2]. This phenomenon aligns with the escalating global threat of AMR, which is rapidly evolving into a major global public health challenge driven by the emergence of multidrug‐resistant (MDR) and pan‐drug‐resistant (PDR) “superbugs” that severely exhaust existing therapeutic options [3]. Among MDR pathogens, *Acinetobacter baumannii* is a leading cause of healthcare‐associated infections and is included in the ESKAPE group of pathogens, which represent a critical global threat to human health due to their ability to cause severe infections and rapidly acquire AMR [4]. In hospital settings, particularly intensive care units (ICUs), *A. baumannii* is frequently associated with ventilator‐associated pneumonia, bloodstream infections, and other invasive infections, predominantly affecting critically ill patients with limited therapeutic options.

Carbapenem resistance in *A. baumannii* arises through multiple mechanisms, including the production of class B, C, and D carbapenemases, decreased membrane permeability, alterations in penicillin‐binding proteins, and overexpression of efflux pumps [5, 6]. Among these mechanisms, the production of carbapenem‐hydrolyzing *β*‐lactamases represents the most clinically significant pathway. In particular, OXA‐type carbapenemases play a central role in resistance and are widely disseminated worldwide [7–9]. The frequent coexistence of OXA‐type carbapenemases with other resistance determinants further restricts therapeutic options available for carbapenem‐resistant *A. baumannii* (CRAB) infections [10]. In addition, NDM‐1 is increasingly reported in combination with *bla*OXA‐23, contributing to broader resistance profiles and severely limiting therapeutic options [11]. Reflecting the clinical and public health importance of this pathogen, the World Health Organization (WHO) classified CRAB as the highest priority pathogen for antibiotic research and development in 2024 [12].

In clinical practice, CRAB isolates are frequently coresistant to multiple antimicrobial classes, severely limiting available therapeutic options. According to the US Centers for Disease Control and Prevention, CRAB caused more than 8000 infections and approximately 700 associated deaths in 2019, underscoring its substantial public health impact [13]. Overall mortality associated with CRAB infections has been reported to exceed 50% in several regions [14]. Hospital‐acquired infections (HAIs) and ventilator‐associated pneumonia caused by *A. baumannii* account for a large proportion of cases, estimated at up to 79.9%, with marked regional variation, ranging from 56.5% in Argentina and 61.8% in Taiwan to nearly 100% in Central America, Pakistan, Lebanon, Qatar, and Croatia. Overall mortality associated with these infections has been reported to reach 56.2% [15]. In Asia, carbapenem resistance rates among *A. baumannii* isolates remain alarmingly high, reaching 71.4% in China and showing widespread predominance of *bla*OXA‐23 in Thailand [16, 17].

In Vietnam, *A. baumannii* is among the three leading causes of HAIs, imposing a substantial burden on public health [18, 19]. Reported carbapenem resistance rates range from 60% to 80% nationwide [20], and infections caused by MDR *A. baumannii* are associated with mortality rates of up to 64% [21]. Although high resistance rates are consistently documented, the distribution of resistance genes varies considerably among healthcare facilities [22–24]. Despite numerous reports on carbapenem resistance in *A. baumannii* in Vietnam, integrated and up‐to‐date studies combining phenotypic antimicrobial susceptibility profiles with detailed carbapenemase genotyping remain limited, particularly in tertiary‐care hospitals where antimicrobial selective pressure is high. The present study is aimed at characterizing the phenotypic resistance profiles and the distribution of carbapenemase‐encoding genes among clinical *A. baumannii* isolates collected from University Medical Center Ho Chi Minh City, to provide updated local data on carbapenem resistance phenotypes and carbapenemase gene distribution in a tertiary‐care hospital setting.

## 2. Methods

### 2.1. Study Location and Collection of Clinical Samples

This is a retrospective study conducted on 156 nonduplicate *A. baumannii* isolates collected from patients at the University Medical Center Ho Chi Minh City (UMC) between January and December 2024, representing respiratory, bloodstream, skin and soft tissue, intra‐abdominal, and urinary tract infections. In addition, isolates obtained from environmental infection control samples were excluded. Isolates that could not be recovered upon subculture or had incomplete stored information were also excluded from the analysis. Demographic and clinical data, including gender, age, infection type by anatomical site, and hospital department, were extracted from electronic medical records and used for subsequent analysis.

All isolates were stored at −80°C and retrieved directly from frozen stocks. They were then revived by subculturing twice onto MacConkey agar (OXOID, United Kingdom) using the streak plate method prior to laboratory analysis to ensure viability and purity. Then, isolates selected for carbapenemase gene detection were preserved and transported to the Vietnam Research and Development Institute of Clinical Microbiology (VCM) in compliance with the three‐layer packaging principle to ensure biosafety during transportation, following the biosafety regulations for the transport of infectious substances issued by the Vietnamese Ministry of Health (Circular 40/2018/TT‐BYT).

### 2.2. Bacterial Identification

Bacterial identification was performed using the BD Phoenix M50 system with BD Phoenix NID identification panels (Becton, Dickinson and Company, United States). A 0.5 McFarland suspension prepared from fresh colonies was inoculated into the NID panel according to the manufacturer′s instructions. Bacterial identification was automatically generated based on biochemical reaction profiles. Quality control was performed using *Escherichia coli* ATCC 25922 in accordance with CLSI M100, 34^th^ edition, guidelines [25].

### 2.3. Antimicrobial Susceptibility Test and Phenotypic Carbapenemase Detection Using Phoenix M50 System

Antimicrobial susceptibility testing (AST) and phenotypic carbapenemase detection in bacterial isolates were performed using the BD Phoenix M50 system with NMIC‐500 panels (Becton, Dickinson and Company, United States). Carbapenemase production was determined using the Phoenix carbapenemase detection algorithm, which classifies isolates as carbapenemase‐producing or non–carbapenemase‐producing based on inhibitor based wells. In detail, a 0.5 McFarland bacterial suspension was prepared, inoculated into Mueller Hinton broth. Bacterial inoculum was poured into the NMIC‐500 panel. The panel was then sealed and inserted into the BD Phoenix M50 system. Susceptibility results were automatically generated within 16–18 h and interpreted as susceptible, intermediate, or resistant according to CLSI M100, 34^th^ edition, breakpoints. Quality control was performed using *Pseudomonas aeruginosa* ATCC 27853 and *E. coli* ATCC 25922 in accordance with CLSI M100, 34^th^ edition, guidelines [25].

### 2.4. Detection of Carbapenemase‐Related Genes by Multiplex Real‐Time PCR Method

Bacterial DNA was extracted using the QIAamp DNA Mini Kit on a QIAcube automated system (QIAGEN, Germany) following the manufacturer′s instructions. Multiplex real‐time PCR was performed to detect specific carbapenemase genes, including *bla*OXA‐23 [26], *bla*OXA‐58 [26], *bla*NDM‐1 [27], and *bla*KPC [27]. Primers and probes were designed by VCM, verified for specificity via NCBI BLAST, and synthesized by SFCprobe Company (Korea) (Table 1). Each 50‐*μ*L reaction mixture contained multiplex master mix and 5.0 *μ*L of DNA template. Amplification was performed on a CFX96 system (Bio‐Rad) under the following conditions: 95°C for 15 min, followed by 40 cycles of 94°C for 15 s and 60°C for 1 min. Appropriate positive and no‐template negative controls were included in each run. Data were analyzed using CFX96 software across FAM, HEX, and Cy5 channels based on amplification plots and Ct values.

**Table 1:** Primer sequences and taqman probes performed in the study.

Gene	Primer	Sequences 5 ^′^➔ 3 ^′^	Reference
*bla*OXA‐23	OXA‐23‐F	GACACTAGGAGAAGCCATGAAG	[26]
	OXA‐23‐R	CAGCATTACCGAAACCAATACG	
	OXA‐23‐P	6‐HEX‐CCAGTCTATCAGGAACTTGCGCGA‐BHQ_1	
			
*bla*OXA‐58	OXA‐58‐F	AAGATTTTACTTTGGGCGAAGC	[26]
	OXA‐58‐R	CAACTTCCGTGCCTATTTGC	
	OXA‐58‐P	Cy5‐TGGACCAATACGACGTGCCAATTCT‐IAbRQSp	
			
*bla*KPC	KPC‐F	GGCCGCCGTGCAAT AC	[27]
	KPC‐R	GCCGCCCAACTCCTTCA	
	KPC‐P	FAM‐TGATAACGCCGCCGCCAATTTGT‐BHQ1	
			
*bla*NDM‐1	NDM1‐F	GACCGCCCAGATCCTCAA	[27]
	NDM1‐R	CGCGACCGGCAGGTT	
	NDM1‐P	HEX‐TGGATCAAGCAGGAGAT‐BHQ1	

*Note:* The multiplex real‐time PCR assay was designed and performed in two separate reaction tubes. Tube 1 contained primers and probes targeting the *bla*KPC and *bla*NDM‐1 genes (along with additional targets, including *bla*OXA‐48 and *bla*GES, included in the commercial kit), with *bla*KPC detected using a FAM‐labeled probe and *bla*NDM‐1 detected using a HEX‐labeled probe. Tube 2 contained primers and probes targeting OXA‐type carbapenemase genes, in which *bla*OXA‐23 was detected using a HEX‐labeled probe and *bla*OXA‐58 using a Cy5‐labeled probe (in addition to other OXA targets such as *bla*OXA‐51 and *bla*OXA‐24 included in the kit). However, in the present study, the analysis focused exclusively on four major carbapenemase genes, namely *bla*KPC, *bla*NDM‐1, *bla*OXA‐23, and *bla*OXA‐58. Therefore, only the corresponding primer and probe sets for these genes are presented.

Abbreviations: BHQ‐1, black hole quencher‐1; *bla*KPC, *Klebsiella pneumoniae* carbapenemase; *bla*NDM‐1, New Delhi metallo‐*β*‐lactamase 1; *bla*OXA‐23, oxacillin hydrolyzing enzyme 23; *bla*OXA‐58, oxacillin hydrolyzing enzyme 58; Cy5, cyanine 5; F, forward; 6‐FAM, 6‐carboxyfluorescein; HEX, hexachloro‐fluorescein; IAbRQSp, Iowa black RQ‐Sp; P, probe; R, reverse.

### 2.5. Study Size and Statistical Analysis

The sample size was calculated using the formula $n\geq {\text{z}}_{12-\alpha /\text{p}\left(1-\text{p}\right)}^{2}/{\text{d}}^{2}$, with a 95% confidence level (*Z* = 1.96), a margin of error (*d*) of 0.05, and an expected prevalence (*p*) of 0.885 based on the prevalence of CRAB reported in a tertiary‐care hospital in India by Shah Kalpesh [28]. This resulted in a required sample size of 156 isolates. Statistical analysis was performed using Stata Version 15.0. Categorical variables, including resistance phenotypes and genotypes, were compared using the Chi‐square or Fisher′s exact test, as appropriate. Univariable logistic regression analysis was applied to evaluate the association between individual factors and carbapenem resistance. The results are expressed as crude odds ratios (OR) and 95% confidence intervals (CI). Statistical significance was set at *p* < 0.05. To minimize bias, only nonduplicate isolates were included, and clinical data extraction followed a standardized protocol.

## 3. Results

### 3.1. Source Characteristics of the Collected Isolates

Among the 156 *A. baumannii* isolates, 110 were identified as CRAB and 46 as CSAB, as presented in Table 2. The CRAB (*n* = 110) and CSAB (*n* = 46) groups exhibited generally comparable baseline characteristics. Patients aged ≥ 60 years accounted for the highest proportion in both groups (65.45% for CRAB and 65.22%, for CSAB). In both groups, respiratory tract infections predominated, accounting for 83.64% of CRAB cases and 65.22% of CSAB cases.

**Table 2:** Demographic and clinical characteristics of carbapenem‐resistant *A. baumannii* (CRAB) and carbapenem‐susceptible *A. baumannii* (CSAB) isolates.

Characteristics	Categories	CRAB (*n* = 110) *n* (%)	CSAB (*n* = 46) n (%)	Crude OR (95% CI)	*p*
Gender	Male	66 (60)	22 (47.83)	1.64 (0.82–3.27)	0.164
	Female	44 (40)	24 (52.17)	1	
					
Age (years)	< 19	13 (11.82)	4 (8.70)	1	
	20–44	10 (9.09)	2 (4.35)	1.54 (0.23–10.15)	0.655
	45–59	15 (13.64)	9 (19.57)	0.51 (0.13–2.06)	0.347
	≥ 60	72 (65.45)	31 (67.39)	0.74 (0.22–2.45)	0.620
					
Infection site	Respiratory tract infections	92 (83.64)	30 (65.22)	1	
	Soft tissue skin infections	10 (9.09)	8 (17.39)	0.41 (0.15–1.13)	0.084
	Bloodstream infections	3 (2.73)	4 (8.70)	0.24 (0.05–1.16)	0.075
	Urinary tract infections	3 (2.73)	3 (6.52)	0.33 (0.06–1.70)	0.184
	Intra‐abdominal infections	2 (1.82)	1 (2.17)	0.65 (0.06–7.45)	0.731
					
Treatment unit	Intensive Care Unit (ICU)	66 (60)	14 (30.43)	5.5 (1.60–18.88)	0.007
	Internal Medicine Department	31 (28.18)	21 (45.65)	1.72 (0.51–5.85)	0.384
	Surgery Department	7 (6.36)	4 (8.70)	2.04 (0.39–10.55)	0.394
	Emergency Department	6 (5.45)	7 (15.22)	1	
					
Source of infection	Hospital‐acquired infection (HAI)	86 (78.18)	23 (50.0)	3.58 (1.72–7.47)	0.001
	Community‐acquired infection (CAI)	24 (21.82)	23 (50.0)	1	
					
Treatment outcome	Improved	73 (66.36)	37 (80.43)	0.73 (0.28–1.88)	0.512
	Recovered	3 (2.73)	1 (2.17)	1.11 (0.10–12.47)	0.935
	Unchanged	19 (17.27)	7 (15.22)	1.0	—
	Worsened	12 (10.91)	0 (0)	NE (perfect prediction)	—
	Severe prognosis—discharged against medical advice (DAMA)	1 (0.91)	1 (2.17)	0.37 (0.02–6.72)	0.500
	Died	2 (1.82)	0 (0)	NE (perfect prediction)	—

*Note:* Crude odds ratios (OR) with 95% confidence intervals (CI) were calculated using binary logistic regression, with the reference category indicated as OR = 1.

Abbreviations: CRAB, carbapenem‐resistant *A. baumannii*; CSAB, carbapenem‐susceptible *A. baumannii*; NE, not estimable due to perfect prediction.

Hospital department was the only factor showing a significant difference between the two groups. Patients admitted to the ICU had a 5.5‐fold higher odds of CRAB infection compared with those treated in other departments (*p* = 0.007). Additionally, HAI was identified as a significant associated factor for CRAB isolation. Patients with HAI had a 3.58‐fold higher odds of CRAB infection than those with community‐acquired infections (CAIs) (*p* = 0.001).

Regarding treatment outcomes, the CSAB group showed high rates of clinical improvement and recovery (80.43% and 15.22%, respectively). In contrast, the CRAB group exhibited a higher proportion of unchanged clinical conditions (17.27%), clinical deterioration (10.91%), and mortality (1.82%). These findings provide an overall characterization of the study population prior to detailed analyses.

### 3.2. AMR Profiles of CRAB and CSAB

Table 3 compares the antimicrobial susceptibility and MIC distributions between CRAB (*n* = 110) and CSAB (*n* = 46) isolates. Overall, CRAB isolates exhibited a severe MDR phenotype, with resistance rates across almost all categories significantly surpassing those of the CSAB group (*p* < 0.001).

**Table 3:** Antimicrobial resistance rates of carbapenem‐resistant *Acinetobacter baumannii* (CRAB) (*n* = 110) and carbapenem‐susceptible *Acinetobacter baumannii* (CSAB) (*n* = 46) isolates.

Antimicrobial agents	Range (*μ*g/mL)	*Acinetobacter baumannii* (*n* = 156)			Carbapenem‐resistant *Acinetobacter baumannii* (*n* = 110)			Carbapenem‐susceptible *Acinetobacter baumannii* (*n* = 46)			*p*
		MIC50 (*μ*g/mL)	MIC90 (*μ*g/mL)	Resistant (%)	MIC50 (*μ*g/mL)	MIC90 (*μ*g/mL)	Resistant (%)	MIC50 (*μ*g/mL)	MIC90 (*μ*g/mL)	Resistant (%)	
Cephalosporins											
Cefotaxim	1– ≥ 64	≥ 64	≥ 64	102 (65.38)	≥ 64	≥ 64	97 (88.18)	16	64	5 (10.87)	< 0.001^a^
Ceftazidime	1– ≥ 64	> 16	> 16	107 (68.59)	> 16	> 16	104 (94.55)	4	8	3 (6.52)	< 0.001^a^
Cefepime	≤ 1– ≥ 64	> 16	> 16	109 (69.87)	> 16	> 16	106 (96.36)	2	8	3 (6.52)	< 0.001^a^
Fluoroquinolones											
Ciprofloxacin	≤ 0.06– ≥ 4	> 2	> 2	100 (64.1)	> 2	> 2	97 (88.18)	0.125	0.5	3 (6.52)	< 0.001^a^
Levofloxacin	≤ 1– ≥ 8	> 4	> 4	98 (62.82)	> 4	> 4	96 (87.27)	≤ 1	≤ 1	2 (4.35)	< 0.001^a^
Aminoglycosides											
Amikacin	≤ 4– > 32	> 32	> 32	85 (55.49)	> 32	> 32	84 (76.36)	8	16	1 (2.17)	< 0.001^a^
Gentamicin	≤ 1– ≥ 16	> 8	> 8	113 (72.44)	> 8	> 8	106 (96.36)	4	> 8	7 (15.22)	< 0.001^a^
Carbapenems											
Imipenem	≤ 0.25– ≥ 16	> 8	> 8	110 (70.51)	> 8	> 8	110 (100)	0.5	1	0 (0)	< 0.001^b^
Meropenem	≤ 0.25– ≥ 16	> 32	> 32	109 (69.87)	> 32	> 32	109 (99.09)	≤ 0.25	0.5	0 (0)	< 0.001^b^
Penicillins + *β*‐lactamase inhibitors											
Ampicillin/sulbactam	≤ 4/2– > 16/8	> 16/8	> 16/8	99 (63.46)	> 16/8	> 16/8	98 (89.09)	≤ 4/2	≤ 4/2	1 (2.17)	< 0.001^a^
Piperacillin/tazobactam	≤ 4/4– ≥ 128	> 64/4	> 64/4	101 (64.74)	> 64/4	> 64/4	101 (91.82)	≤ 4/4	16/4	0 (0)	< 0.001^b^
Folate pathway antagonists											
Trimethoprim/sulfamethoxazole	≤ 0.5/9.5– ≥ 320	> 2/38	> 2/38	99 (63.46)	> 2/38	> 2/38	94 (85.45)	≤ 0.5/9.5	> 2/38	5 (10.87)	< 0.001^a^
Tetracyclines											
Minocycline	≤ 1–16	≤ 1	8	11 (7.05)	≤ 1	8	11 (10.0)	≤ 1	≤ 1	0 (0)	0.010^b^

Abbreviation: MIC, minimum inhibitory concentration.

^a^Chi‐square test.

^b^Fisher test.

Within the CRAB cohort, resistance to carbapenems was nearly absolute. Resistance rates reached 100% for imipenem and 99.09% for meropenem, with both MIC50 and MIC90 values exceeding the resistance breakpoints (> 8 *μ*g/mL for imipenem and > 32 *μ*g/mL for meropenem). Extensive coresistance was also observed among noncarbapenem antimicrobial classes. Resistance rates ranged from 88.18% to 96.36% for cephalosporins, ≥ 87.27% for fluoroquinolones, and 96.36% for gentamicin. Similarly, high resistance rates were identified for ampicillin/sulbactam (88.09%) and piperacillin/tazobactam (91.82%), accompanied by elevated MIC90 values of > 16/8 and > 64/4 *μ*g/mL, respectively. In contrast, CSAB isolates remained highly susceptible to most tested noncarbapenem agents, with resistance rates generally below 16% (*p* < 0.001). Among the tested agents, minocycline demonstrated the highest in vitro activity. It retained complete susceptibility against CSAB isolates (MIC90 ≤ 1 *μ*g/mL) and showed the lowest resistance rate among CRAB isolates (10.0%, MIC90 = 8 *μ*g/mL; *p* = 0.010).

**Figure 1:**
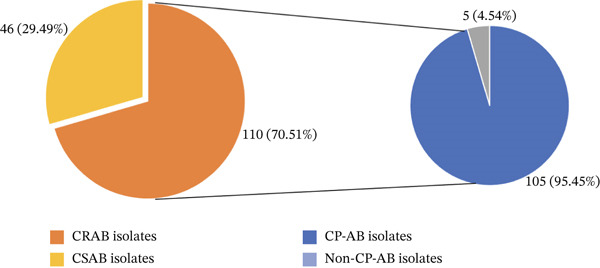
Prevalence of carbapenemase production among carbapenem‐resistant *A. baumannii* isolates. *The primary chart shows the overall distribution of carbapenem-resistant A. baumannii (CRAB) (* *n* = 110 *) and carbapenem-susceptible A. baumannii (CSAB) (* *n* = 46 *) isolates among the total A. baumannii collection (* *n* = 156 *). The secondary chart shows the distribution of carbapenemase-producing A. baumannii (CP-AB) (* *n* = 105 *) and non–carbapenemase-producing A. baumannii (non–CP-AB) (* *n* = 5 *) isolates within the CRAB group.*

Among the 156 *A. baumannii* isolates, 70.51% (110/156) were identified as CRAB (Figure 1). Phenotypic carbapenemase testing of the 110 CRAB isolates showed that the majority were carbapenemase‐producing *A. baumannii* (CP‐AB), accounting for 95.45% (105/110). In contrast, 4.54% (5/110) of CRAB isolates exhibited carbapenem‐resistant phenotypes despite negative phenotypic carbapenemase detection results.

### 3.3. Distribution of Carbapenemase Genes in CRAB Isolates

**Figure 2:**
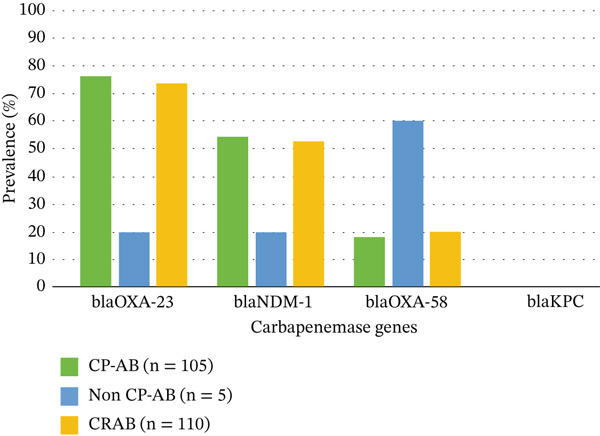
Distribution of carbapenemase genes among carbapenemase‐producing *Acinetobacter baumannii* (CP‐AB, *n* = 105), non–carbapenemase‐producing *Acinetobacter baumannii* (non–CP‐AB, *n* = 5), and carbapenem‐resistant *Acinetobacter baumannii* (CRAB, *n* = 110) isolates. *blaOXA-23—oxacillin hydrolyzing enzyme 23, blaOXA-58—oxacillin hydrolyzing enzyme 58, blaNDM1—New Delhi metallo-β-lactamase 1, blaKPC—Klebsiella pneumoniae carbapenemase.*

Among the 110 CRAB isolates, *bla*OXA‐23 was the most prevalent carbapenemase gene (73.6%), followed by *bla*NDM‐1 (52.7%) and *bla*OXA‐58 (20.0%). The *bla*KPC genes was not detected (Figure 2). Moreover, a similar distribution was observed in the carbapenemase producing *A. baumannii* (CP‐AB) subgroup (*n* = 105), with detection rates of 76.2% for *bla*OXA‐23, 54.3% for *bla*NDM‐1, and 18.1% for *bla*OXA‐58. In contrast, the non–carbapenemase‐producing *A. baumannii* (non–CP‐AB) subgroup (*n* = 5) exhibited low detection rates overall, except for *bla*OXA‐58, which was detected in 60.0% of isolates.

**Figure 3:**
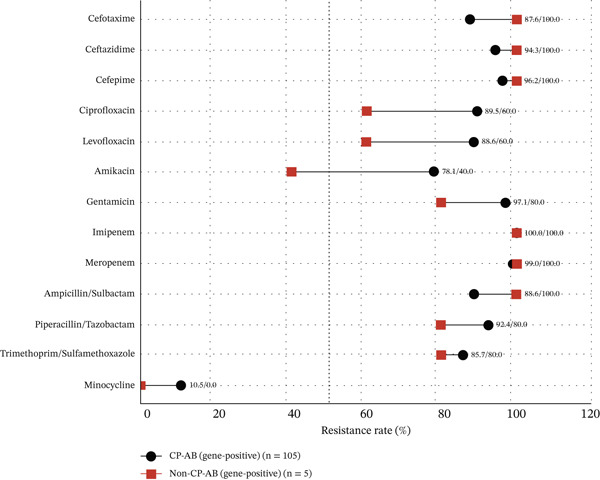
Antimicrobial resistance profiles of carbapenemase‐producing (*n* = 105) and non–carbapenemase‐producing *A. baumannii* (*n* = 5) isolates (Fisher′s exact test, *p* > 0.05).

Five non–CP‐AB isolates carrying resistance genes were also identified. Although resistance rates appeared numerically higher in several antimicrobial classes compared with carbapenemase‐producing isolates, these differences were not statistically significant (*p* > 0.05) (Figure 3).

**Figure 4:**
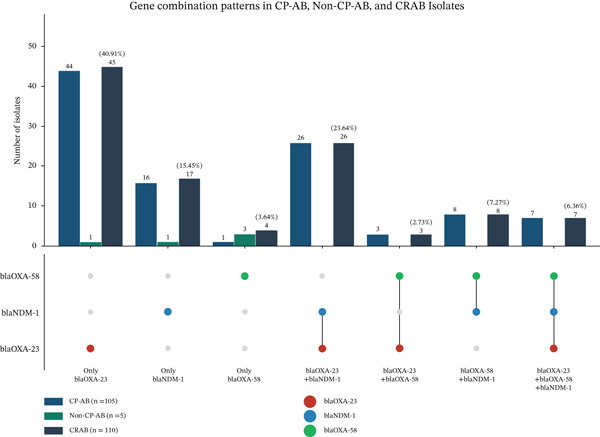
Gene combination patterns identified among carbapenemase‐producing *A. baumannii* (CP‐AB, *n* = 105), non–carbapenemase‐producing *A. baumannii* (non–CP‐AB, *n* = 5), and carbapenem‐resistant *A. baumannii* (CRAB, *n* = 110) isolates.

The distribution and combination patterns of the detected carbapenemase genes among the 110 CRAB isolates are shown in Figure 4. Overall, a single carbapenemase gene was identified in 60% (66/110) of the isolates, with *bla*OXA‐23 being the most prevalent standalone gene 40.91% (45/110), followed by *bla*NDM‐1 15.45% (17/110) and *bla*OXA‐58 3.64% (4/110). Notably, gene co‐existence was observed in 40.00% (44/110) of the CRAB isolates. Two gene combinations accounted for 33.64% (37/110) of the isolates, predominantly driven by *bla*OXA‐23 + *bla*NDM‐1 (23.64%). A highly similar distribution was observed within the CP‐AB subgroup, where 58.10% harbored a single gene, 35.24% carried two genes, and 6.67% possessed all three genes. Overall, the vast majority of isolates possessed either one or two carbapenemase genes, with the co‐existence of *bla*OXA‐23 and *bla*NDM‐1 representing the most prevalent combination.

**Figure 5:**
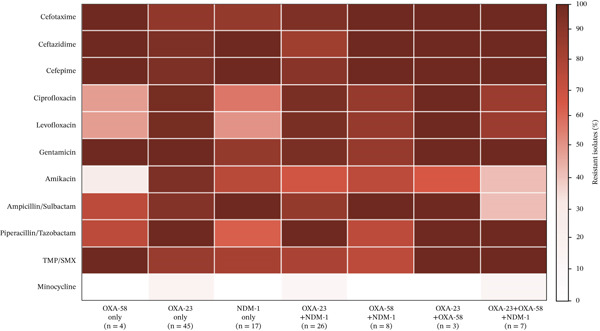
Descriptive heat map showing antimicrobial resistance rates among CRAB isolates stratified by carbapenemase genotype. *Color intensity represents the percentage of resistant isolates within each genotype group. The figure illustrates genotype-specific resistance patterns and was not designed to infer clonal relatedness or molecular clustering among isolates (* *n* = 110 *).*

Consistent with this distribution, Figure 5 showed differences in resistance phenotypes across carbapenemase genotype groups. Isolates carrying *bla*OXA‐23 or *bla*NDM‐1, particularly in combination, exhibited high and relatively uniform resistance levels across multiple antimicrobial classes. In contrast, isolates harboring *bla*OXA‐58 alone showed more heterogeneous resistance patterns. Overall, isolates carrying multiple carbapenemase genes tended to demonstrate broader resistance phenotypes than isolates carrying a single carbapenemase gene.

## 4. Discussion

This study provides an overview of the epidemiological and clinical characteristics of CRAB and CSAB isolates collected at a tertiary‐care hospital. Overall, baseline patient characteristics were comparable between the two groups, with patients aged ≥ 60 years, representing the largest proportion in both cohorts. This finding is consistent with previous reports identifying older adults as a vulnerable population for *A. baumannii* infections [29, 30]. Respiratory tract infections were the predominant source of isolation in both groups, especially among CRAB isolates (83.64%), reflecting the well‐established association between *A. baumannii* and ventilator‐associated pneumonia in ICU settings [30, 31]. However, no statistically significant difference in infection type was observed between CRAB and CSAB, suggesting that the anatomical site of infection alone is not a determining factor for carbapenem resistance.

In contrast, treatment unit was strongly associated with the odds of CRAB isolation. The proportion of CRAB isolates recovered from the ICU (60%) was higher than that reported in most countries in Europe [32, 33] and North America [34]. Patients admitted to the ICU had a 5.5‐fold higher odds of CRAB infection compared with those treated in other wards. Similar findings were reported by Sheng et al. (2010), who demonstrated a 3.42‐fold higher odds of CRAB infection (95% CI: 1.76–5.26; *p* = 0.008), which increased to 6.12‐fold in cases of bloodstream infection (95% CI: 1.58–13.68; *p* = 0.009) [35]. Likewise, Zhang et al. (2023) reported that invasive procedures (OR = 9.41) and prior carbapenem exposure (OR = 2.54) significantly increased the odds of CRAB infection in pediatric patients [36]. Collectively, these findings indicate that the ICU represents a setting characterized by intense antibiotic selective pressure, frequent invasive interventions, and a high risk of MDR bacterial transmission [37]. In addition, HAI was identified as an important factor associated with CRAB, with a 3.58 fold higher odds compared with CAI. This observation is consistent with regional and global surveillance data, showing that CRAB is predominantly detected in hospital environments and remains uncommon in the community settings [38, 39]. The prolonged environmental persistence of *A. baumannii* and its ability to spread via cross‐transmission are considered key drivers of CRAB dissemination within healthcare facilities. Although the CRAB group exhibited a trend toward poorer clinical outcomes, including higher rates of clinical deterioration and mortality, these differences could not be formally estimated or statistically evaluated (not estimable) due to the absence of such adverse events in the CSAB group. Nevertheless, this trend is consistent with previous reports indicating that CRAB infections are associated with worse prognosis and increased treatment burden [35, 39].

With respect to carbapenem resistance, the study demonstrated marked differences in AMR phenotypes between CRAB and CSAB isolates. The predominance of CRAB (70.51%) indicates a substantial burden of carbapenem resistance at the study site (Figure 1), consistent with reports from Vietnam and Southeast Asia, where CRAB typically accounts for 60%–80% of *A. baumannii* isolates in tertiary‐care hospitals [16, 20]. The CRAB isolates exhibited a severe multidrug resistance phenotype, with near‐absolute resistance to the carbapenem imipenem (100% resistance, both MIC50 and MIC90 > 8 *μ*g/mL) and meropenem (99.09% resistance, both MIC50 and MIC90 > 32 *μ*g/mL), and very high resistance rates to third and fourth‐generation cephalosporins, aminoglycosides, and fluoroquinolones (Table 3). The ATLAS surveillance program (2012–2019) analyzed 2674 *A. baumannii* isolates from 13 countries in the Asia Pacific region and reported an average carbapenem resistance rate of 71.7%, ranging from 2.8% in Japan and 6.5% in Australia to 83%–88% in Thailand, Pakistan, India, and South Korea [40]. Within the specific CRAB cohort evaluated by the ATLAS program, resistance to meropenem was absolute at 100%, with both MIC50 and MIC90 values reaching 32 *μ*g/mL, whereas imipenem exhibited a resistance rate of 99.2%, with an MIC50 and MIC90 of 16 *μ*g/mL [40]. These international benchmarks demonstrate that high‐level resistance and dramatic upward shifts in MIC profiles are well‐established phenotypic traits of CRAB populations in the region, providing a critical reference point for the high resistance rates and elevated MICs identified in our local hospital setting. In South Korea (2022), carbapenem resistance among CRAB isolates reached 99.1%–99.5%, accompanied by high resistance rates to cephalosporins (97.2%–98.6%), aminoglycosides (70.7%–77.8%), and fluoroquinolones (97.2%) [41]. In Thailand (2021), the majority of MDR *A. baumannii* isolates were 100% resistant to carbapenems, with additional resistance to ceftazidime (99.23%), gentamicin (91.85%), amikacin (82.96%), and ciprofloxacin (97.78%) [17]. In Vietnam, a study by Ta et al. (2025) reported that CRAB isolates exhibited 100% resistance to third‐ and fourth‐generation cephalosporins, 97.7%–100% resistance to aminoglycosides, and 100% resistance to fluoroquinolones [42]. These findings suggest that CRAB has become an endemic healthcare‐associated pathogen across multiple regional tertiary‐care centers. Ultimately, this profound MDR phenotype characterized by near‐universal carbapenem resistance underscores the urgent need for enhanced surveillance, strict infection control, and optimized antimicrobial stewardship in clinical practice. Notably, minocycline retained the highest in vitro activity against CRAB isolates in the present study, with a resistance rate of only 10.0% and a relatively low MIC90 value (8 *μ*g/mL). Similar findings have been reported in previous surveillance studies, in which minocycline demonstrated preserved activity against MDR *A. baumannii*, including CRAB isolates [43, 44]. These findings suggest that minocycline may remain a potential therapeutic option for selected CRAB infections, particularly in settings with limited treatment alternatives. However, the clinical efficacy of minocycline should be interpreted cautiously because in vitro susceptibility may not always correlate with clinical outcomes. In the present study, 4.54% (5/110) of CRAB isolates showed carbapenem‐resistant phenotypes despite negative phenotypic carbapenemase detection results (Figure 1). Similar findings have been reported previously by Shahraki et al., who identified 4% (5/123) of CRAB isolates without detectable carbapenemase production [45]. These observations suggest that carbapenem resistance in *A. baumannii* may not be solely associated with phenotypic carbapenemase production and may involve additional resistance determinants that were not evaluated in the present study [46].

In the analysis of carbapenemase gene distribution, *bla*OXA‐23 was the predominant carbapenemase genotype, detected in nearly three‐quarters of CRAB isolates (Figure 2). This finding is consistent with previous studies reporting OXA‐type carbapenemases, particularly *bla*OXA‐23, as among the most prevalent carbapenem resistance determinants in CRAB isolates worldwide [47]. Joshi et al. (2017) reported the presence of *bla*OXA‐23 in all CRAB isolates examined [48], whereas Zhang et al. (2021) observed that 93.4% of CRAB isolates carried *bla*OXA‐23 [49]. Similar distributions have also been reported in other Asian countries. In Thailand (2021), most MDR *A. baumannii* isolates were dominated by class D *β*‐lactamases, with *bla*OXA‐23 detected in 92.59% of isolates, whereas *bla*NDM‐1 (4.44%) and *bla*OXA‐58 (1.48%) were detected at much lower frequencies [17]. Likewise, a study from India reported that 97.7% of CRAB isolates harbored *bla*OXA‐23‐like genes, whereas the prevalence of *bla*NDM‐1 (29.1%) and *bla*OXA‐58 (3.5%) remained substantially lower [11]. Collectively, these findings indicate that *bla*OXA‐23 remains a predominant carbapenemase genotype among CRAB isolates in both regional and global settings. In parallel, *bla*NDM‐1 has been increasingly reported in *A. baumannii* globally, with high occurrence in India and China [50]. The presence of blaNDM‐1 demonstrates a pattern of resistance to all beta‐lactam agents except monobactams, including last‐resort carbapenems [51]. In Vietnam, NDM‐type carbapenemase was first detected in Gram‐negative pathogens in 2010 [52]. In the study, 52.7% of MDR *A. baumanni* isolates were found to carry NDM‐type carbapenemase (Figure 2), and these isolates were collected at tertiary hospitals, indicating the spread of drug‐resistant isolates in Vietnam.

Beyond the predominant carbapenemase‐producing CRAB population, a distinct subset of isolates lacking phenotypic carbapenemase activity warrants further consideration. In our study, among carbapenem‐resistant but non–CP‐AB isolates, the detection frequencies of *bla*OXA‐23 and *bla*NDM‐1 were relatively low (20% each), whereas *bla*OXA‐58 was identified in 60% of isolates, suggesting a potential contributory role of *bla*OXA‐58 in this context. Previous research by Deniz Gur et al. in Turkey emphasized the importance of transcriptional regulation in drug resistance expression of the *bla*OXA‐58 gene without the mechanism of carbapenemase production [53]. Therefore, the observed discrepancy between genotype and phenotype suggests that carbapenem resistance in these isolates may be influenced by regulatory mechanisms affecting blaOXA‐58 expression, underscoring the multifactorial nature of resistance in *A. baumannii.* In addition, 20% (1/5) of non–CP‐AB isolates harbored *bla*NDM‐1. Although these isolates did not demonstrate phenotypic carbapenemase production, resistance genes were still detected genotypically, suggesting that additional resistance mechanisms may contribute to the observed carbapenem‐resistant phenotype. Moreover, non–CP‐AB isolates showed high resistance rates across several antimicrobial classes, comparable to those observed in CP‐AB isolates (Figure 3). However, these differences were not statistically significant (*p* > 0.05), likely due to the limited sample size of the non–CP‐AB subgroup. In line with the genotypic findings, most CRAB isolates harbored one or two carbapenemase genes (Figure 4), indicating the presence of diverse resistance determinants among CRAB. Among the detected genes, *bla*OXA‐23 was the predominant standalone carbapenemase gene (40.91%). This finding is consistent with previous studies conducted in Asia and worldwide, which have identified OXA‐23 as the major carbapenemase associated with carbapenem resistance in clinical *A. baumannii* isolates [49, 54].

The co‐existence of multiple carbapenemase genes was also frequently observed. In particular, the combination of *bla*OXA‐23 and *bla*NDM‐1 was the most common two‐gene pattern, in agreement with previous reports in Vietnam [59]. The concurrent detection of OXA‐type carbapenemases and metallo‐*β*‐lactamases may be associated with broader resistance profiles among CRAB isolates. Although less common, isolates carrying the three‐gene combination (*bla*OXA‐23, *bla*OXA‐58, and *bla*NDM‐1) were also identified. A similar pattern has been reported in a tertiary‐care hospital study from Thailand, although at a lower frequency (0.9%) [55]. The presence of isolates harboring multiple carbapenemase genes may reflect the increasing molecular diversity of CRAB isolates in hospital settings. A comparable distribution was observed in the CP‐AB subgroup, in which most isolates also carried one or two carbapenemase genes. These findings suggest that the coexistence of multiple carbapenemase determinants is not uncommon among CRAB isolates in clinical settings.

Building on the distribution of carbapenemase genes, the present study showed that AMR phenotypes varied according to the detected genotypic patterns (Figure 4). CRAB isolates carrying *bla*OXA‐23 or *bla*NDM‐1, either alone or in combination, generally exhibited high resistance rates to most tested antimicrobial agents (Figure 5). This finding is biologically plausible because OXA‐type carbapenemases and metallo‐*β*‐lactamases are well‐recognized contributors to carbapenem resistance in *A. baumannii*, with *bla*OXA‐23 being one of the most widely disseminated carbapenemase genes globally [56]. Isolates harboring *bla*NDM‐1 alone also showed high resistance to several non–*β*‐lactam agents in the present heat map. However, because this study only assessed selected carbapenemase genes and phenotypic susceptibility, this pattern should be interpreted as an observed association rather than evidence of a specific underlying mechanism. Previous reviews have noted that NDM‐producing *A. baumannii* is often reported in MDR or extensively drug‐resistant isolates, but the resistance phenotype may depend on the broader genetic background of each strain [57].

In contrast, isolates carrying *bla*OXA‐58 alone demonstrated a more heterogeneous resistance profile, with comparatively lower resistance rates to amikacin, ciprofloxacin, and levofloxacin. This finding is consistent with reports suggesting that OXA‐58 may be associated with variable carbapenem resistance levels and that its phenotypic expression can differ among isolates [58]. Importantly, isolates carrying multiple carbapenemase genes, particularly *bla*OXA‐23 + *bla*NDM‐1 with or without *bla*OXA‐58, demonstrated more uniform and extensive resistance across the tested antimicrobial agents. Similar findings have been reported in previous studies describing broader MDR phenotypes among *A. baumannii* isolates coharboring OXA‐type and NDM carbapenemases [56, 57]. However, as the present study only evaluated phenotypic antimicrobial susceptibility, the specific genetic determinants responsible for this coresistance to non–*β*‐lactam agents could not be definitively established.

This study has several limitations. The relatively small sample size and limited number of isolates in several subgroups may have reduced the precision of some OR estimates, as reflected by the wide CI; therefore, these findings should be interpreted with caution. In addition, five CRAB isolates exhibited carbapenem‐resistant phenotypes despite negative phenotypic carbapenemase detection, although resistance genes were identified genotypically, suggesting that additional resistance mechanisms may be involved [54]. Molecular analyses such as whole‐genome sequencing, MLST, and characterization of virulence‐associated factors were not performed. Future studies incorporating next‐generation sequencing may help clarify the molecular epidemiology and resistance mechanisms of CRAB isolates. Despite these limitations, the present study provides valuable local data on the phenotypic and genotypic characteristics of CRAB isolates at a major tertiary‐care referral hospital in southern Vietnam. Given the high patient burden and geographically diverse referral population at the study hospital, these findings may provide useful insights into local CRAB epidemiology and AMR trends in clinical practice.

## 5. Conclusion

This study demonstrated a high prevalence of CRAB in a Vietnamese tertiary‐care hospital, characterized by extensive multidrug resistance and a predominance of *bla*OXA‐23, frequently co‐occurring with *bla*NDM‐1. Most CRAB isolates exhibited phenotypic carbapenemase production; however, a subset of isolates showed carbapenem‐resistant phenotypes despite negative phenotypic carbapenemase detection results, suggesting the possible involvement of additional resistance mechanisms. The observed association between CRAB isolation, ICU stay, and HAI highlights the important role of healthcare settings in the persistence and dissemination of MDR *A. baumannii*. Collectively, these findings underscore the need for continuous phenotypic and genotypic surveillance, strengthened infection prevention and control measures, and optimized antimicrobial stewardship strategies to limit the spread of CRAB in clinical settings.

## Author Contributions

**T.H.**: conceptualization, methodology, data analysis, writing – original draft, writing – review and editing, supervision. **Q.C.**: conceptualization, methodology, data collection, data analysis, writing – original draft. **L.L., K.N., D.B., T.P.:** resources, data collection and analysis. **M.D.J.**: data analysis, writing – review and editing. **V.P.**: conceptualization, methodology, resources, data collection.

## Funding

This study was supported by the Research and Development, 10.13039/100006190, Research Contract No. 66/2025/HĐ‐DHYD.

## Disclosure

All authors read and approved the manuscript.

## Ethics Statement

This study was approved by the Institutional Review Board (IRB)/Biomedical Research Ethics Committee of the University Medical Center Ho Chi Minh City (Approval No. 91/GCN‐HĐĐĐ, June 30, 2025).

## Consent

All authors have read the manuscript and consent to publish.

## Conflicts of Interest

The authors declare no conflicts of interest.

## Data Availability

The data that support the findings of this study are available from the corresponding author upon reasonable request.
